# Non-immune immunoglobulins shield *Schistosoma japonicum* from host immunorecognition

**DOI:** 10.1038/srep13434

**Published:** 2015-08-24

**Authors:** Chuang Wu, Nan Hou, Xianyu Piao, Shuai Liu, Pengfei Cai, Yan Xiao, Qijun Chen

**Affiliations:** 1 MOH Key Laboratory of Systems Biology of Pathogens, Institute of Pathogen Biology, Chinese Academy of Medical Sciences & Peking Union Medical College, Beijing 100730, P.R. China; 2 Key Laboratory of Zoonosis, The Ministry of Education, Jilin University, Changchun 130062, P.R. China

## Abstract

Schistosomiasis is a major human parasitic disease with a global impact. *Schistosoma japonicum*, the most difficult to control, can survive within host veins for decades. Mechanisms of immune evasion by the parasite, including antigenic variation and surface masking, have been implicated but not well defined. In this study, we defined the immunoglobulin-binding proteomes of *S. japonicum* using human IgG, IgM, and IgE as the molecular bait for affinity purification, followed by protein identification by liquid chromatography with tandem mass spectrometry (LC-MS/MS). Several proteins situated at the tegument of *S. japonicum* were able to nonselectively bind to the Fc domain of host immunoglobulins, indicating a mechanism for the avoidance of host immune attachment and recognition. The profile of the immunoglobulin-binding proteomes provides further clues for immune evasion mechanisms adopted by *S. japonicum*.

Schistosomiasis, also known as bilharzia, is an acute and chronic parasitic disease caused by blood flukes of the genus *Schistosoma*. It is the second most devastating parasitic disease after malaria according to worldwide rates of both morbidity and mortality. In 2012, at least 249 million people and a large number of animals were infected in 78 countries, and an estimated 700 million people were at risk (WHO, February 2014). Schistosomiasis japonica is caused by parasitization with *Schistosoma japonicum* and is the only zoonotic schistosomiasis; therefore, it is the most difficult to control among the five schistosome species that infect humans^1^. Currently, praziquantel is the only drug that is effective for the treatment of the disease, and a large amount of work is still required to develop a vaccine that is able to control the disease effectively^2^.

The surface of the schistosome tegument represents the most accessible interface for a host immune attack and therefore merits investigation for vaccine candidates. Nevertheless, although vaccines based on the membrane components or associated proteins have been extensively studied, little success has been achieved^2^,^3^,^4^. The fact that adult parasites can thrive within the mesenteric or vesicular veins of their hosts for decades indicates that the worms employ a variety of strategies to escape identification by the immune system. It has been hypothesized that schistosomal parasites evade host immune expulsion through surface masking, molecular mimicry, and the active modulation of host immune responses^5^. A variety of host molecules, such as immunoglobulins, major histocompatibility complex products, complement components, and blood group antigens, have been found on the surface of the worms^5^,^6^,^7^,^8^. For example, the heavy chains of host IgM, IgG1, and IgG3 plus the α-chain of the C3c/C3dg fragment of complement component C3 have been found on the tegumental surface of *S. mansoni*^7^,^9^. Although the binding regions of the Igs and the mechanism by which the parasite inactivates C3 have not been characterized^10^, the acquisition of host components on its surface is believed to benefit the parasite by preventing host recognition.

To date, only a few schistosomal membrane proteins have been confirmed as the ligands for host immunoglobulins, and most of these studies have focused on *S. mansoni*. For example, the non-filamentous paramyosin (SCIP-1) associated with the schistosomula of the parasite membrane of both *S. mansoni* and *S. japonicum* has been proposed both as a Fc receptor of a nonspecific host IgG and as an inhibitor of complement C9 polymerization^6^,^11^,^12^,^13^,^14^. Sjc23, a member of the tetraspanin (TSP) family of *S. japonicum*, mediated the acquisition of human IgG via the interaction of a nine amino acid motif with the Fc domain of the IgG molecule^15^; the specific antibodies after immunization with Sjc23 were predominantly the IgG2a type, which has been shown to be inefficient in complement fixation and to exhibit fewer cytophilic properties in ADCC (antibody-dependent cell-mediated cytotoxicity)^16^.

In this study, to investigate the host-schistosome interaction and the mechanism of immune evasion employed by *S. japonicum*, we applied immobilized versions of the non-immune human immunoglobulins G, M and E as molecular baits to selectively precipitate parasite interactive components that were subsequently identified by LC-MS/MS. The immunoglobulin binding proteomes of adult *S. japonicum* were obtained and analyzed carefully. Our results will facilitate the rational design of vaccines based on schistosomal tegument-associated antigens.

## Results

### Host immunoglobulins are abundantly observed on the surface of *S. japonicum*

**Figure 1:**
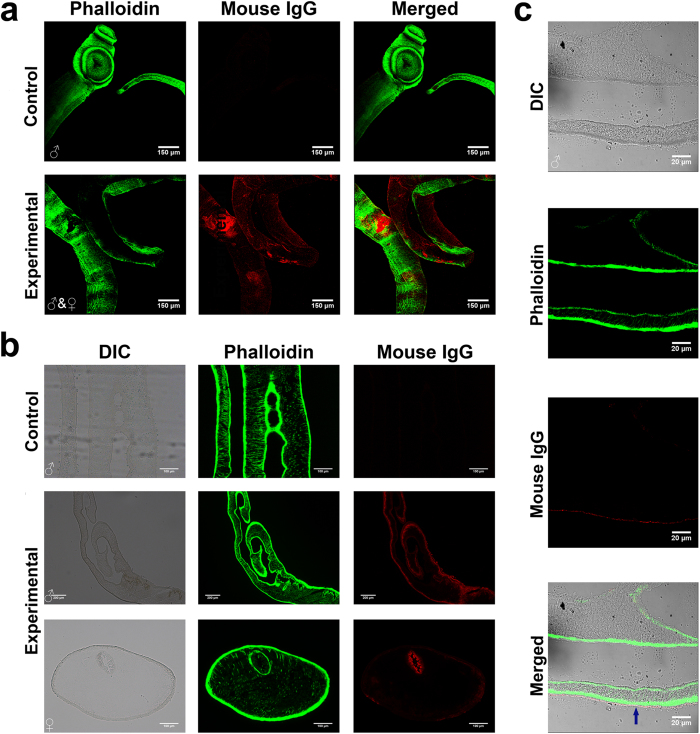
Detection of mouse IgG binding on the surface of murine adult *S. japonicum*. (**a**) Detection of mouse IgG on the surface of whole adult worms using laser confocal microscopy. F-actin was labeled with Alexa Fluor 488 phalloidin (green) and mouse IgG was detected with Alexa Fluor 555 donkey anti-mouse IgG (red) on the surface of both male (right) and female (left) adult worms. (**b**) Detection of mouse IgG (in red) on the surface of frozen sections of schistosome using fluorescence microscopy. (**c**) Detection of mouse IgG (in red) on the surface of a frozen section of adult blood flukes using laser confocal microscopy. DIC: differential interference contrast.

**Figure 2:**
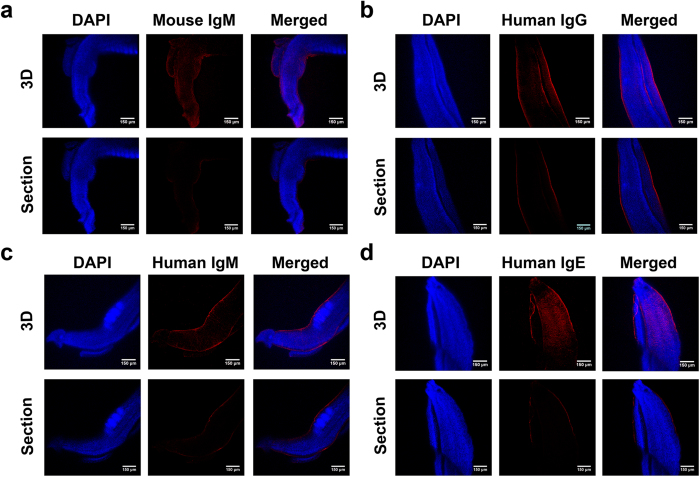
Detection of mouse IgM and human IgG, IgM and IgE binding to the surface of adult worms under laser confocal microscopy. (**a**)Detection of mouse IgM (in red) on the surface of whole adult worms (stained in blue). (**b**–**d**) Detection of the binding of human IgG, IgM, and IgE to whole adult worms using IFA (indirect immunofluorescent assay), respectively. The red fluorescence signals indicate binding of the immunoglobulin to the parasites. In each image group, the upper panel represents a 3D view of the worm, and the lower panel represents a sectional image of one of the serial sections.

To detect the binding of non-immune IgG and IgM on the surface of adult *S. japonicum*, IgG- and IgM-specific antibodies conjugated with a red fluorophore were separately incubated with either parasites directly flushed from the veins of infected mice or parasite sections. Mouse IgG was detected on the surface of both male and female intact adult worms using a laser confocal microscopy (Fig. 1a). The fluorescence visualized using fluorescence microscopy was more intense on the surface of male worms compared with that of females (Fig. 1b). Indeed, imaging of frozen sections using the laser confocal microscopy showed that the fluorescence signals were located on the outermost layer of the blood fluke (Fig. 1c). Additionally, mouse IgM was also observed on the parasite surface (Fig. 2a), and the adsorption of human IgG, IgM and IgE on the parasite surface was clearly observed (Fig. 2b–d). No cross reactivity was observed with the secondary fluorescence-labeled antibodies (data not shown).

### Identification of 437 *S. japonicum* proteins with affinity to human non-immune immunoglobulins

**Figure 3:**
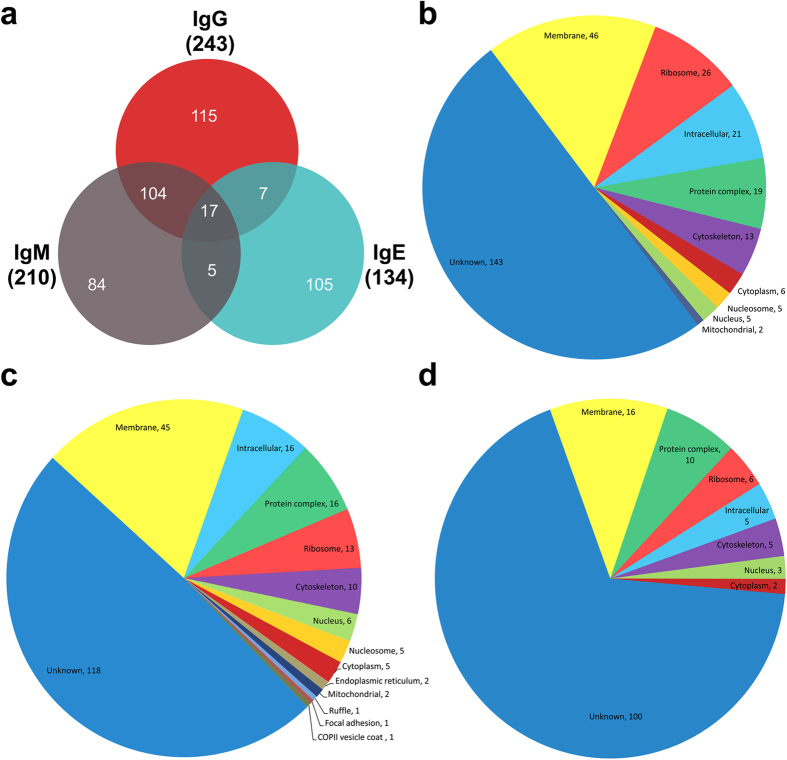
Proteomic analyses of immunoglobulin-binding proteins of *S. japonicum*. (**a**) Venn diagram showing the overlapping numbers of proteins that bound to each of the non-immune human immunoglobulins. (**b**–**d**) Gene ontology (GO) analysis of the putative cellular components of the IgG, IgM and IgE-binding proteomes of *S. japonicum*.

To identify the protein interactions with the host non-immune Igs, native proteins were extracted from adult *S. japonicum* and separately incubated with human IgG-, IgM-, or IgE-conjugated Sepharose beads. The bound proteins were eluted and analyzed by LC-MS/MS following trypsinization. The MS/MS spectra were searched against the *S. japonicum* protein database. Only the proteins detected more than twice within three replications that were not identified among the retrieved proteins in the control group were classified as part of their corresponding Ig-binding proteome. In total, 437 non-immune human immunoglobulin-binding proteins of *S. japonicum* were identified, with 243 proteins bound to IgG, 210 bound to IgM and 134 bound to IgE. Several proteins showed affinity for both IgG and IgM, while the proteins that bound IgE were much more selective (Fig. 3a). The entire list of the 437 proteins is cataloged in Supplementary Data 1.

Next, the proteins in the three Ig-binding proteomes were functionally categorized. The protein sequences were annotated with Gene ontology (GO) terms in three independent categories: cellular components (Fig. 3b–d), molecular functions (Supplementary Fig. 1), and biological processes (Supplementary Fig. 2). In the cellular component category, a major proportion was predicted to be involved with the membrane (Fig. 3b–d). For those proteins associated molecular functions, the majority were annotated with enzyme activity, protein binding, and ion binding. The biological functions and sequence features of the identified proteins were assigned using BLASTp against annotated proteins in the GenBank database, and the transmembrane proteins were predicted using the TMHMM engine. The categories of proteins that were abundant in the proteome list or may have potential significance are presented in Table 1.

**Figure 4:**
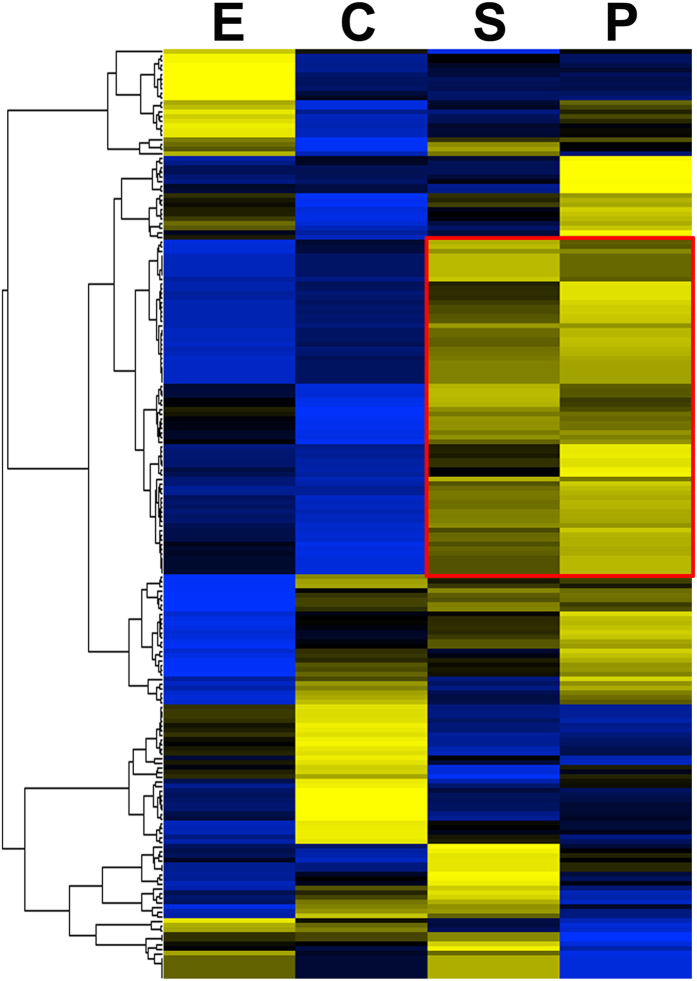
Hierarchical clustering of the expression profiles of the *S. japonicum* immunoglobulin-binding proteins during four developmental stages. The heat-map of 200 genes encoding for the potential Ig-binding proteins extracted from the microarray dataset (E, eggs; C, cercariae; S, hepatic schistosomula; P, paired adult worms). Most of the genes were up-regulated in the cercariae, schistosomula or adult worms. Genes in the red box represent a cluster of genes abundantly expressed both in the schistosomula and adult worms while the schistosome lives in the definitive host. The color scale represents the relative expression levels, with yellow indicating an up-regulated gene, blue a down-regulated gene, and black a gene with unchanged expression.

Gene expression analysis using an oligonucleotide microarray was performed to measure the expression patterns of the Ig-binding proteins during the four parasite developmental stages in rabbits and in the adult stage of four distinct animal hosts. The heatmap shown in Fig. 4 illustrated that most of the genes encoding for the Ig-binding proteins were predominantly expressed while the schistosomes lived in their definitive host. Genes in the red box represent a cluster of genes highly expressed both in the schistosomula and in adult worms. Only four proteins were differentially expressed among the animal hosts (Supplementary Table 1).

### Confirmation of the immunoglobulin-binding behavior of the identified proteins

**Figure 5:**
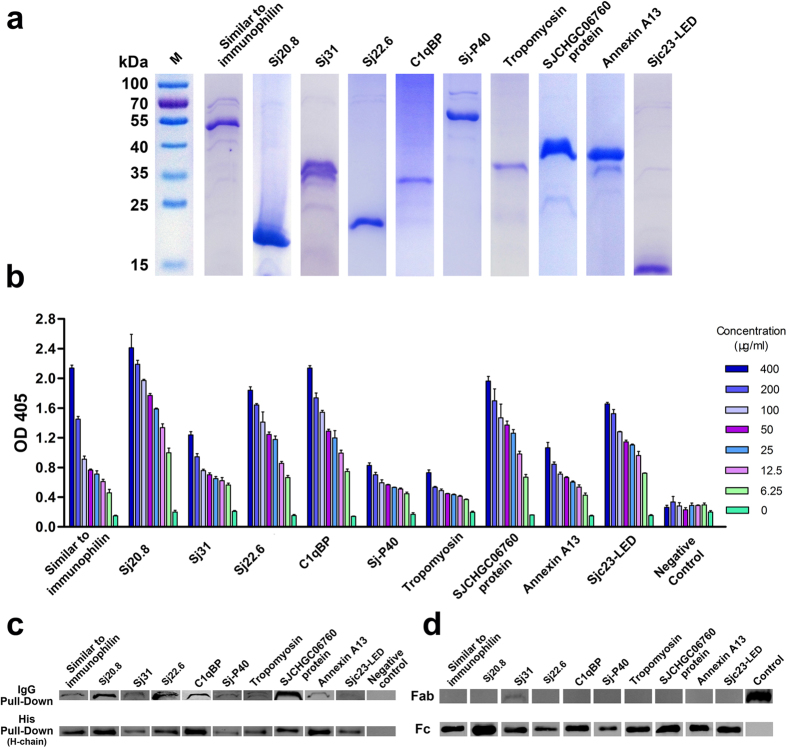
Binding characterization of the ten schistosomal proteins with non-immune human immunoglobulin G. (**a**) SDS-PAGE confirmation of ten His-tagged recombinant proteins. (**b**) Binding of the ten proteins with human non-immune IgG using an ELISA assay. Purified human plasma IgG from individuals without schistosomiasis was applied to ELISA plates and used to evaluate the affinity of ten proteins for it. All ten proteins showed capacity for binding to human IgG. (**c**) Binding of the ten proteins with human non-immune IgG in the pull-down assay. Human IgG-Sepharose and His-tagged proteins immobilized on Ni-NTA agarose resin were used as bait to capture the fusion protein or human IgG, respectively. The affinity was detected by Western blot using the appropriate antibodies. The binding capacity of the ten proteins to human IgG was confirmed using both the IgG pull-down and His pull-down assays. (**d**) The binding of human IgG domains to the ten proteins in the pull-down assay. The Fab and Fc fragments of human IgG were incubated with fusion protein-conjugated particle. After washing, the binding was detected by Western blot. Sjc23-LED was used as a positive control for binding with the Fc fragment and a negative control for binding with the Fab fragment. An unrelated His-tagged protein was used as a negative control for the Fc fragment, and the purified Fab fragment was used as a positive loading control for detection. All ten proteins bound to the Fc fragment, while only Sj31 marginally precipitated the Fab fragment with a dim band.

To confirm the Ig-binding behavior of the proteins identified using a proteomic approach, we selected 10 proteins with features of possible surface exposure and predicted Ig-binding properties (Table 2) and recombinant proteins were generated (Fig. 5a). Their interaction with Ig was analyzed using a classical ELISA assay and pull-down approach. In the ELISA assay, the ten His-tagged recombinant proteins bound to non-immune human IgG, while the unrelated His-tagged protein did not exhibit any binding activity (Fig. 5b). Furthermore, the recombinant parasite proteins were able to be pulled down by IgG, and the immobilized recombinant proteins were able to precipitate human IgG as well (Fig. 5c).

### *S. japonicum* proteins only interact with the Fc domain of human Ig

To investigate the interaction between the *S. japonicum* proteins and the host non-immune Ig and to identify the molecular region of human IgG that binds the *S. japonicum* proteins, the Fab and Fc fragments of human IgG were incubated with recombinant proteins immobilized on Sepharose beads. Only the Fc fragment was precipitated by all of the recombinant proteins; no adhesion to the Fab fragment was observed except with recombinant Sj31 (Fig. 5d). The binding between the Fc fragment and the fusion proteins was also confirmed using an ELISA assay (Supplementary Fig. 3).

### Affinity of *S. japonicum* proteins for various immunoglobulin isotypes

There are five antibody isotypes in placental mammals: IgA, IgD, IgE, IgG, and IgM. These isotypes are classified by their heavy chains and are denoted by the Greek letters α, δ, ε, γ, and μ. Schistosomiasis japonica is the only parasitic zoonosis among all forms of schistosomiases. Hence, we tested the binding capacity of the *S. japonicum* proteins to immunoglobulins isolated from human and animal species using an ELISA assay. The results are summarized in Table 3. For human IgM and IgE, the ELISA results were the same as those of the affinity pull-down assays. Of the 10 schistosomal proteins, only four showed affinity for IgA and five bound to IgD. These proteins displayed similar affinities to porcine and bovine IgG as to human IgG, with the exception of Sjc23, which did not exhibit any affinity for bovine IgG.

**Figure 6:**
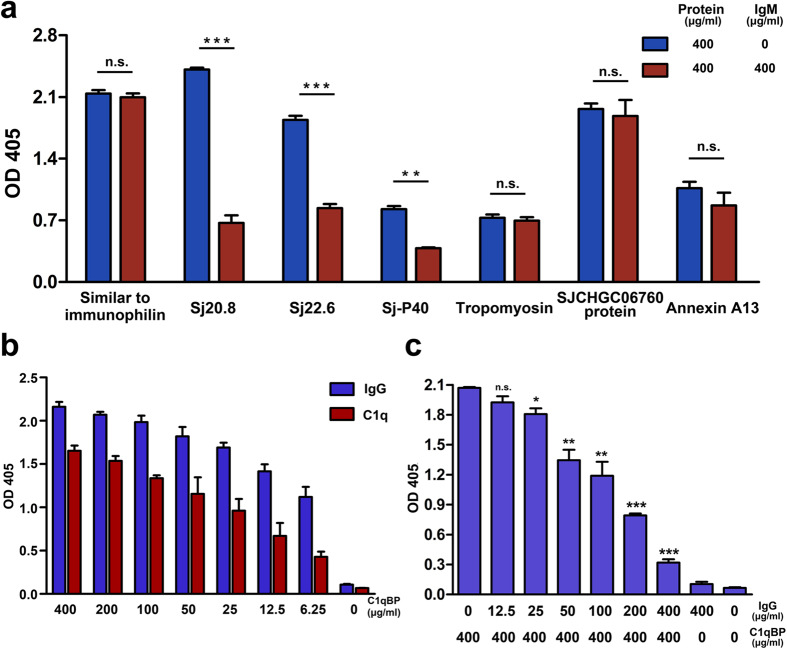
The competitive binding between various Igs or Ig/complement factors with schistosomal ligands. (**a**) The competitive binding between IgG and IgM with seven proteins that bound to both types of immunoglobulins. The ELISA plates were coated with human IgG, and recombinant proteins were mixed with the same amount of human IgM by weight and then added to the wells. The OD_405_ values were compared with those of wells without the addition of IgM (mean ± s.d. of three independent experiment replicates, *P < 0.05, **P < 0.01, n.s., not significant, two-tailed Student’s *t*-test). (**b**) Comparison of the binding of C1qBP with human C1q and IgG by ELISA. Plates were coated with equal amounts of human IgG and C1q by weight and incubated with doubling diluted recombinant C1qBP. C1qBP bound to both human IgG and C1q, and the OD values for the binding to human IgG were even higher than those for C1q. (**c**) The inhibitory effect of human IgG on the binding between complement and C1qBP was investigated using an ELISA assay. ELISA plates were coated with C1q prior to incubation with C1qBP mixed with human IgG at various concentrations (mean ± s.d. of three independent experiment replicates, *P < 0.05, **P < 0.01, ***P < 0.001, n.s., not significant, two-tailed Student’s *t*-test). IgG significantly competed with the binding of C1q to C1qBP.

Seven of the ten selected recombinant proteins exhibited an affinity for both human IgG and IgM. Therefore, we performed a competitive binding assay to confirm whether the host Igs interacted with the same site on the schistosomal proteins. We coated ELISA plates with human IgG, followed by the addition of fusion proteins mixed with an equal amount of human IgM to the wells; then, the OD_405_ values of the wells with or without the addition of IgM were compared. As shown in Fig. 6a, the measurements collected for wells of three of the proteins (Sj20.8, Sj-P40 and Sj22.6) containing IgM were significantly different (*P* < 0.01) from those of wells lacking IgM. IgM did not show any inhibitory effects on the interaction of the remaining 4 proteins with human IgG. These results implied that the latter four proteins use unique sites to bind human IgG and IgM, while the former three proteins either use the same sites or exhibit steric hindrance between the two sites.

### C1qBP binds to human IgG more strongly than to C1q

According to the result of BLASTp using the protein sequences as query sequences, we found that protein AAW26757.1 contains the same sequence as the functional domain of CAX76877.1, which has been predicted with C1q binding capacity by Zhou *et al.*^17^. To confirm the interaction between the complement C1q binding protein (C1qBP) with complement C1q and human IgG, we coated the wells of a microtiter plate with equal amounts of C1q or human IgG and then incubated with doubling diluted recombinant C1qBP. As displayed in Fig. 6b, similar dose-dependent binding relationship between C1q and C1qBP was observed for C1qBP-human IgG. However, the affinity between C1qBP and human IgG was much stronger than that between C1q and C1qBP. Next, we tested the inhibitory effect of human IgG on the C1q-C1qBP interaction. ELISA plates were coated with human C1q and incubated with C1qBP mixed with various concentrations of human IgG. As depicted in Fig. 6c, IgG significantly inhibited the C1q-C1qBP interaction (*P* < 0.001), even at low concentrations.

### The human immunoglobulin-binding proteins are predominantly expressed during the adult stage of the parasite

**Figure 7:**
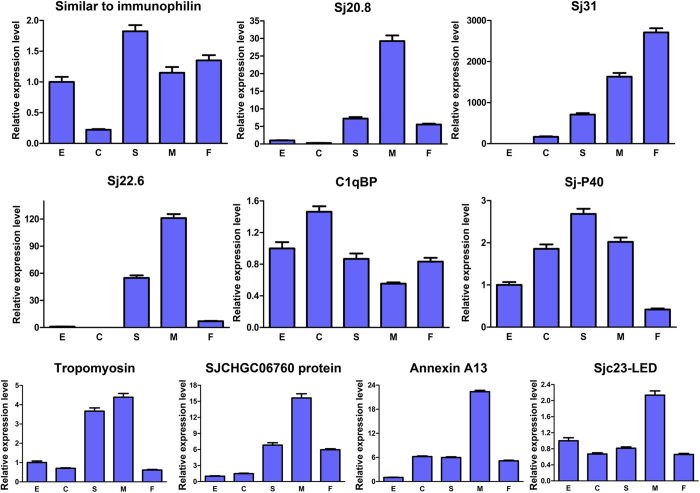
Expression analysis of genes encoding the ten immunoglobulin-binding proteins using quantitative real-time PCR. The expression levels of the ten Ig-binding proteins were validated for various developmental stages by relative qRT-PCR analysis. The relative expression levels of the genes were calculated using SDS v1.4 software (Applied Biosystems), and the error bars represent the standard deviation for three technological replicates. (E, eggs; C, cercariae; S, hepatic schistosomula; M, adult male worms; F, adult female worms).

We investigated the expression patterns of the ten proteins during various developmental stages of the parasite using qRT-PCR. In general, most of the genes were expressed predominately in male parasites during the late developmental stages (Fig. 7). For five of the genes (encoding proteins Sj20.8, Sj22.6, SJCHGC06760 protein, Annexin A13, and Sjc23), the expression levels in adult male worms were at least twice those in worms at other stages, while the tropomyosin gene was also highly expressed in the hepatic schistosomula.

## Discussion

The draft genome sequence of *S. japonicum* has been completed and has provided valuable clues for understanding parasite biology and host-parasite interactions^17^. The pathology of schistosomiasis has been mainly attributed to constant egg deposition in the liver of infected individuals. Thus, elimination of the parasites in the veins is the optimal way to cure the disease. It has been known that adult worms can thrive in the mesenteric or vesicular veins for decades. However, although surface masking and antigenic variation have been implicated, the mechanism is unclear. In this study, we used immunofluorescence to illuminate the surface of worms processing affinity for both mouse and human immunoglobulins. Then, we affinity-purified the proteins with host non-immune immunoglobulins^18^. Of the 437 schistosomal proteins identified, 243 bound to human IgG, 210 to human IgM, and 134 to human IgE; most of the proteins showed affinity for both IgG and IgM, while fewer showed affinity for IgE (Fig. 3a and Supplementary Data 1). Furthermore, most of the proteins could be categorized based on sequence features, including EF-hand motif, immunoglobulin motif, Ca^2+^ binding motif, annexin superfamily, immunophilin, and complement binding motif, suggesting common properties of immunoglobulin binding among the proteins identified. Even though a number of proteins were predicted to be surface-exposed proteins, some of them might be exposed after the washing in the citrate buffer. For example, Sj22.6 was detected in the apical cytoplasm of the tegument of male and female worms of *S. japonicum* by immunofluorescence in an earlier report^19^. Thus further investigation are needed to definitively localize the proteins with uncharacterized features.

To confirm and further characterize the interactions of the schistosomal proteins with the host immunoglobulins, ten proteins were selected (Table 2), and recombinant proteins were generated. The binding of these proteins with host immunoglobulins was confirmed using both an ELISA assay and a pull-down approach. Compared with ELISA assays, pull-down assays require higher affinity between the ligand and receptor due to the high stringency of particle precipitation and the associated washing steps. All of the selected proteins showed a high affinity for the IgGs of both humans and domestic animals and variable but reduced affinity for IgM, IgE and IgA (Fig. 5 and Table 3). Previous studies on the antigen paramyosin of *S. mansoni*^6^,^11^,^12^,^13^,^14^ and Sjc23^15^ suggested that these proteins only adhere to the Fc domain of Ig; in agreement with this result, we found that all of the tested recombinant proteins bound the Fc domain except Sj31, which showed a slight affinity for the Fab domain (Fig. 5d and Supplementary Fig. 3). It is likely that one of mechanisms schistosomal parasites escape host recognition is through binding to the Fc domain of immunoglobulins. One consequence of this Fc domain binding is that the reaction of complement activation and fixation is paralyzed due to the reduced availability of the paratope-epitope interaction of the Fab domain with the antigen, resulting in the unavailability of the complement binding domain. Furthermore, the complement receptor protein identified in the parasite (C1qBP) exhibited higher affinity for human IgG than for complement C1q, strongly suggesting that the parasite utilized non-immune Ig to block complement activation.

To further confirm the importance of these proteins for interactions with the host immune system, we investigated their expression during various developmental stages of the parasite using qRT-PCR. The results showed that they were mainly expressed during the stages associated with the infection of their definitive hosts.

The identification of the immunoglobulin-binding proteome of *S. japonicum* not only confirmed the findings reported earlier but also extended the understanding regarding the function of the surface localized proteins of the parasite. For example, Sjc23, a member of the tetraspanin protein family, is a 23 kDa surface-exposed protein of *S. japonicum* that has been regarded as a potential candidate for a schistosomiasis vaccine. However, in our previous study we found that this protein elicited rapid humoral responses mediated by the IgG2a subclass after infection and did not provide protection against challenge with cercariae^16^. It was further determined that Sjc23 could bind to human non-immune IgG through the interaction of a 9-amino acid motif (-KIQTSFHCC-) with the Fc domain of human IgG^15^. This binding may be responsible for the rapid but biased immune response after immunization with the recombinant Sjc23 protein or parasite infection^16^. Hence, it was demonstrated that Sjc23 is a schistosomal molecule with IgG-binding properties that facilitates parasite immune regulation in a manner similar to that of paramyosin. In the current study, Sjc23 and paramyosin were enriched and identified, validating the coverage and veracity of the affinity-based purification approach.

The immunoglobulin superfamily (IgSF) is a large group of cell surface and soluble proteins involved in the recognition, binding or adhesion processes of cells. Members of the IgSF exhibit characteristic immunoglobulin-like folding, which is defined by two opposing antiparallel β-sheets connected in a unique manner^20^,^21^,^22^. Some of the secreted or cell surface-localized members of the IgSF have been suggested to play central roles in regulating the adaptive and innate immune responses. An essential activity of the ectodomains of these proteins is the specific recognition of diverse cognate ligands^23^. For example, many members of the IgSF, including FcγRIIB, PIR-B, BTLA, PD-1 and KIR, have been defined as inhibitory receptors that mediate immunosuppression via binding to their specific ligands. Previous studies have shown that viruses and other pathogens that engage IgSF receptors contribute to the selection of humoral mediators of adaptive immunity^21^,^22^,^23^. However, the function of the schistosomal IgSF members and their interaction with the mammalian host immune system is still not understood. Here, the finding that this family of proteins selectively recognize and bind to host immunoglobulins, which are their homologous proteins, which suggests that they may lead to suppressive immunoreaction, and contribute to the inhibition of anti-schistosoma immunity as observed in other pathogens such as adenovirus, coronavirus, and reovirus^20^.

Tegument proteins with EF-hand (EFh) domains composed of two perpendicular alpha helices separated by a loop to form a Ca^2+^-binding site^24^ were another group shown to exhibit immunoglobulin-binding properties. Although proteins with EF-hand domains are commonly found in the cytoplasm and membranes^25^, they are rarely found in the tegument membrane, with the exception of trematodes, such as *S. japonicum*^26^,^27^, *S. mansoni*^28^, *S. haematobium*^29^, *F. hepatica*^30^, *F. gigantica* and *C. sinensis*^31^. Here, 9 EF-hand domain-containing proteins were identified that possessed human Ig-binding capacity, and this finding was confirmed by further studies with recombinant proteins. The results suggested that EF-hand domain-containing tegument proteins perform specific functions in trematodes by interacting with host immune molecules.

Parasite proteases contribute to pathogenesis in a variety of ways, including invasion, nutrition acquisition, immune evasion, and host-parasite interactions^32^,^33^. M28, a 28 kDa membrane serine protease of *S. mansoni*, cleaves iC3b and can restrict attack by effector cells that utilize complement receptors (especially CR3); treatment with protease inhibitors can potentiate the killing of schistosomula by complement plus neutrophils^34^,^35^,^36^. It has been reported that the schistosomula stages of *S. mansoni* release an elastase-like serine protease that degrades IgE in both humans and rodents but not IgG in humans^37^. Proteases secreted by *T. cruzi* trypomastigotes can enzymolyze the Fc fragment of normal IgG, suggesting that the FcRs inhibit the cytotoxic effects of the classical pathway^38^,^39^. Furthermore, a fraction (PSA-Fc^+^) isolated from the suckers and tegument of *E. granulosus* protoscoleces was described to possess proteolytic activity specific for human IgG1 and IgG3 through Fc binding^40^. In the present study, we identified six cathepsins in *S. japonicum* that exhibited an affinity for human Igs. Specifically, Sj31, a cathepsin B-like cysteine proteinase, was confirmed to possess an affinity for human, porcine, and bovine IgG and human IgD. Similar to other Ig-binding proteins identified in this study, its binding target region is also the Fc domain of IgG, which supports its role in immunoevasion.

Tropomyosin (Tpm) has been described as an invertebrate “panallergen”^41^ in species ranging from shellfish and dust mites to the parasitic nematode *Anisakis simplex*^42^,^43^. Similar to paramyosin, Tpm is aconstitutive protein of the muscular tissue in schistosomes and is located in the worm epidermis^44^. Due to the numbers of tropomyosin isoforms in schistosomes, it is likely that Tpm induces a range of responses in the host. In a recent report analyzing SmTpm, 12 of 20 predicted splice variants from the four SmTpm genes of *S. mansoni* were detected, and four variants of TpmII (TpmII.4, 8, 3 and 7) were analyzed. The authors concluded that TpmII.3 interacted with IgE and that TpmII.7 interacted with IgG_4_^45^. Here, we identified a variant of SjTpm that interacted with human, porcine and bovine IgG, and human IgM. Another study by our laboratory identified 25 predicted splice variants from the five SjTpm genes^46^; however, identifying their target components and understanding their association with the immune response requires further studies.

Annexin, which is also known as lipocortin due to its phospholipase A2 suppressive activity, is a common name for a group of cellular proteins found in eukaryotic organisms. Although they are cytosolic proteins that lack signal sequences, they have also been detected in extracellular fluids and are associated with cell surface membranes where they could be involved in anti-hemostatic and anti-inflammatory functions. Schistosomal annexins have previously been identified on the tegumental surface of the parasite and as excretory/secretory products by immuno-electron microscopy in both the schistosomula and adult stages of *S. mansoni* and *S. bovis*^47^,^48^,^49^. However, their functions are still unclear. Nine of the annexin proteins were found to be enriched in this study and the affinity of two members for host Igs was confirmed, implying a more cryptic function in immunoregulation.

The complement system is an important component of the innate immune response and can be recruited and brought into action by the adaptive immune system. This system helps or “complements” the ability of antibodies and phagocytic cells to clear pathogens from an organism. C1q is a glycoprotein that belongs to the collectin family and has the largest molecular weight among all complement molecules. C1q is the first component of the classical pathway of complement activation. The globular head of C1q exclusively binds to the CH2 domain of IgG molecules or to the CH3 domain of IgM to alter its conformation, leading to activation of the C1r and C1s proteases and the classical complement pathway. However, this activation occurs only after immunoglobulin binding in the form of immune complexes. Here, we verified that C1qBP could bind to both C1q and human IgG, with a stronger affinity for the latter than for the former. Moreover, human IgG could efficiently block the binding of C1q to C1qBP, potentially inhibiting complement activation and the classical complement pathway. Our real-time PCR results showed that C1qBP was expressed during all developmental stages of the parasite and was most abundant during the cercaria stage, implying that C1qBP may play a key role in protecting the juvenile parasites from immune attacks in the early period after invasion and maintaining this effect during other parasitic stages.

In summary, our findings propose that human immunoglobulins can interact with multiple *S. japonicum*-derived ligands and consequently have profound inhibitory effects on anti-schistosome immune responses. Our data are the first to provide a global view of the interaction between the schistosomal parasite and human non-immune immunoglobulins, suggesting a molecular basis for the surface masking and evasion of immune recognition after invasion.

## Methods

### Parasite samples

All experimental protocols were approved by the Animal Care and Use Committee of the Institute of Pathogen Biology of the Chinese Academy of Medical Sciences & Peking Union Medical College (IPB-2011-6). The methods were carried out in accordance with the approved guidelines.

Freshly shed wild-type cercariae of *S. japonicum* were harvested from infected *Oncomelania hupensis* collected from the Dongting Lake region, Hunan Province, China. New Zealand rabbits and Balb/c mice were infected with 400 or 40 cercariae, respectively, via the skin of the abdomen. After 42 days of infection, adult worms were washed out from the hepatic portal vein using perfusion methods^50^. The rabbit-hosted worms were rinsed with PBS buffer (HyClone, Logan, USA) containing a protease inhibitor cocktail (Sigma, CA, USA) and divided into single-use aliquots (approximately 150 mg, wet weight). The samples were then snap-frozen and stored in liquid nitrogen until use. Male and female schistosomes obtained from infected mice were manually detached under light microscopy and host proteins adsorbed on the surface were detached by washing in 100 mM sodium citrate in PBS buffer. A portion of the separated worms were dehydrated according to a standard protocol^50^,^51^ and stored at −20 °C until use. The rest were processed into frozen sections: the worms were embedded within Tissue OCT-Freeze Medium, and cut into slices (7 μm thick) at −20 °C using freezing microtome (SELL, London).

### Immunofluorescence assay

Mouse IgG binding on the tegumental surface of *S. japonicum* was detected using an immunofluorescence assay with both intact whole worms and frozen sections. For the whole worms, the assay was based on the standard protocol^51^ with several minor modifications. Briefly, the whole worms were fixed in 4% formaldehyde and then treatment with proteinase K (2 μg/ml) for 10 min followed by permeabilized for 2 h with 1% SDS in PBS buffer at room temperature. After permeabilization, the parasites were re-fixed for 10 min in 4% formaldehyde and rinsed in PBSTx buffer (0.3% Triton X-100 in PBS buffer) and then incubated in blocking solution (5% horse serum, 0.05% Tween-20 in PBSTx buffer) for 2 h at room temperature. The blocked worms were incubated with Alexa Fluor 555 donkey anti-mouse IgG (H+L) and Alexa Fluor 488 phalloidin at 4 °C overnight. After washing four times (30 minutes each) in the dark, the samples were mounted in permanent aqueous mounting medium (AbD Serotec, Kidlington, UK), and the fluorescence was visualized with a TCS SP5 confocal microscope (Leica Microsystems, Wetzlar, Germany). Parasites treated with sodium citrate were assayed as controls.

For the frozen sections, the samples were fixed for 5 minutes in 4% formaldehyde at room temperature, followed by three washes with PBS buffer. We then proceeded to the blocking step using the method described above, and images were obtained with both a fluorescence microscope (Nikon, Tokyo, Japan) and a laser confocal microscope.

For the detection of mouse IgM on the parasite surface, whole worms were treated as described above and then stained with a combination of DAPI and Alexa Fluor 555 donkey anti-mouse IgM (μ chain).

For detection of the binding capabilities of human Igs, freshly isolated whole worms were extensively washed in PBS containing 100 mM sodium citrate to strip off murine Igs from their surfaces. The worms were incubated separately with human IgG, IgM (Sigma, CA, USA) and IgE (Abcam, Cambridge, UK) and then treated as described above. The worms were stained with a combination of DAPI and Alexa Fluor 594 goat anti-human IgG (H+L), Alexa Fluor 594 goat anti-human IgM (μ chain) or TRITC-conjugated goat anti-human IgE (ε chain). All fluorescent antibodies and dyestuffs were purchased from Invitrogen (Oregon, USA).

### Affinity purification of native *S. japonicum* proteins with immobilized nonimmune human immunoglobulin G, M and E

Human IgG, IgM and IgE were covalently coupled to CNBr-activated Sepharose 4B (GE Healthcare, Uppsala, Sweden) and blocked with Tris-HCl according to the operation manual.

Frozen parasite samples were homogenized by grinding in liquid nitrogen, followed by incubation with PBS buffer containing an EDTA-free protease inhibitor cocktail (Sigma, CA, USA) for 15 min with gentle shaking at 4 °C. Ultrasonic disruption was used for 3 s and then repeated 80 times at 6 s intervals on ice. The mixtures were shaken at 4 °C for 30 min and centrifuged at 18,000 r/min for 30 min to remove tissue and cell debris. The protein concentration was quantified using the BCA kit (Thermo, Waltham, USA) in accordance with the manufacturer’s instructions.

Approximately 10 mg of protein was diluted to 0.5 mg/ml in TST buffer (50 mM Tris, 150 mM NaCl, and 0.05% Tween-20, pH7.6) and incubated with 100 μl IgG-, IgM- or IgE-Sepharose for 4 h with gentle agitation. The beads were washed ten times in large volumes of TST buffer followed by two more washing steps using 1 ml of 5 mM NH_4_Ac (pH5.0). The bound proteins were eluted with 200 μl elution buffer (0.5 M HAc, adjusted to pH3.4 with NH_4_Ac). The Sepharose resin was used as a control for nonspecific adsorption to the resin. All affinity purification steps were performed at 4 °C.

### Mass spectrometry analyses

Proteins eluted from the beads were first reduced for 1 h at 56 °C by adding dithiothreitol (DTT) to reach a concentration of 10 mM and then alkylated with 55 mM iodoacetamide (IAM) at room temperature for 45 min in the dark, followed by trypsinization at 37 °C for 16 h. The LC-MS/MS analysis was performed with an ACQUITY UPLC system (Waters, Milford, USA) coupled to a TripleTOF 5600 mass spectrometer (AB Sciex, Framingham, MA, USA). Briefly, the samples were analyzed using an RP C_18_ capillary LC column (70 μm × 150 mm, 3 μm) from Michrom BioResources (Auburn, USA). The eluted gradient was 5–30% buffer B (99.9% acetonitrile/0.1% formic acid) at a flow rate of 500 nl/min for 60 min. The mass spectrometer was used to analyze the samples. The MS data were acquired in a high sensitivity mode using the following parameters: 30 data-dependent MS/MS scans per each full scan; full scans were acquired at a resolution of 40,000 and MS/MS scans at 20,000; 35% normalized collision energy, charge state screening (including precursors with +2 to +4 charge states) and dynamic exclusion (exclusion duration 15 s); and the MS/MS scan range was 100–1,800 m/z with a scan time was 100 ms.

The MS/MS spectra were searched against the *S. japonicum* protein database (12,657 sequences, 4,929,382 residues) downloaded from the Chinese Human Genome Center at Shanghai (CHGC, http://lifecenter.sgst.cn/schistosoma/en/schdownload.do) using Mascot software version 2.3.02 (Matrix Science, London, UK). Trypsin was chosen as the cleavage specificity with a maximum number of allowed missed cleavages of two. The searches were performed using a peptide and product ion tolerance of ±0.05 Da. Scaffold was used to further filter the database search results using the decoy database method. The following filters were used in this study: a 1% false positive rate at the protein level and at least 2 unique peptides contained in each protein.

### Bioinformatics analyses

Only proteins detected more than two times within three biological replicates were included in the following analyses. Putative functions and sequence features were assigned using BLASTp against annotated proteins in the GenBank database, and the alignment was classified to be significant if the corresponding *E* values < 1E-5. The transmembrane proteins were predicted using TMHMM Server v.2.0 (http://www.cbs.dtu.dk/services/TMHMM/)^52^. InterPro domains were annotated by InterProScan (Release 27.0), and functional assignments were mapped onto Gene Ontology (GO)^53^,^54^,^55^.

### Gene expression analysis

An oligonucleotide microarray was used to measure the expression patterns of the obtained Ig-binding proteins at four developmental stages (eggs, cercariae, hepatic schistosomula and adult worm pairs) in rabbits and at the adult stage in various hosts (rabbits, C57/BL6 mice, Balb/c mice and buffalo). The design and construction of the microarray, and the methods used for microarray hybridization and feature extraction have been previously reported^56^. Raw data and normalized gene level data from the array have been deposited in the public database Gene Expression Omnibus (http://www.ncbi.nlm.nih.gov/geo/) under accession numbers for the platform GPL18617, and series GSE57143. Local BLAST searches were performed to identify the microarray sequences that corresponded to Ig-binding protein sequences used as query sequences. Genes were considered to be significantly differentially expressed with expression fold-changes ≥2 between any two compared stages or hosts and *P*-values < 0.05 (one-tailed Student’s *t*-test). All of the genes encoding the Ig-binding proteins expressed in the adult stage and a total of 200 genes differentially expressed during four developmental stages and 4 genes differentially expressed in four hosts were selected according to the above criteria. Hierarchical clustering analysis of the 200 genes was performed to generate a heatmap using Cluster 3.0 software and Heatmap Builder 1.0 software^57^.

### Recombinant protein expression

In addition to Sjc23-LED, 9 non-immune Ig-binding proteins were selected for further confirmation analyses. The genes of these ten proteins were amplified from the cDNA of adult *S. japonicum* using high-fidelity Phusion DNA polymerase (Finnzymes Oy, Vantaa, Finland). The amplified fragments were cloned into the pET-22b or pET-32a vectors (Novagen, San Diego, CA, USA) and then solubly expressed in *E.coli* (Transgen, Beijing, China). The fusion proteins were purified by His GraviTrap columns (GE Healthcare, Uppsala, Sweden) according to the protocol provided by the manufacturer. The characteristics of these proteins, primer sequences, vectors and *E.coli* competent cells are presented in Table 2.

### Confirmation of the binding of the selected proteins with non-immune immunoglobulins by ELISA and pull-down assays

For the ELISA, we coated plate wells with five human immunoglobulins (IgG, IgM (Sigma, CA, USA), IgE (Abcam, Cambridge, UK), IgA, and IgD (Calbiochem, Darmstadt, Germany) and two animal host IgGs (porcine and bovine IgGs from Sigma) (250 ng/well) overnight at 4 °C. His-tagged proteins were added to the wells in a series of dilutions ranging from 400 μg/ml to 6.25 μg/ml and then incubated for 1 h at 37 °C. An uncorrelated His-tagged protein (Sjsp-13, AAP05892.1) was used as a negative control. The binding was detected with His-tag mouse McAb (Abmart, Shanghai, China) and ALP-anti-mouse-IgG antibody (Sigma, CA, USA) at OD_405_.

A pull-down assay was also performed to confirm the specific binding between these proteins and human IgG. The IgG pull-down was performed following a procedure similar to that of the affinity purification of *S. japonicum* proteins with human Igs. Aliquots of the retrieved proteins were resolved in 12% SDS-PAGE and blotted onto polyvinylidene difluoride (PVDF) membranes. The binding was visualized with anti-His McAb and IRDye 800 CW-conjugated goat anti-mouse IgG (H+L) antibody (Li-COR Biosciences, Lincoln, NE, USA) using Odyssey (Li-COR). For the His pull-down, the His-tagged protein immobilized on Ni-NTA agarose resin (QIAGEN GmbH, Hilden, Germany) was used as a bait protein and incubated with human non-immune IgG as previously described^15^,^58^. Briefly, 100 μg of His-tagged protein was incubated with 100 μl Ni-NTA Sepharose resin for 30 min at room temperature. After washing to remove the unbound proteins, 100 μg of human IgG was mixed with the recombinant protein-bound resin and incubated at 4 °C for 2 h with gentle agitation. GST-bound-glutathione sepharose resin was used as a negative control. After incubation, the beads were washed ten times with 1.2 ml PBST buffer (0.05% Tween-20 in PBS buffer) to remove the unbound proteins. The proteins were then eluted in 100 μl 1.5 M NaCl by gentle agitation at 4 °C for 1 h. The binding was detected as described above with IRDye 800 CW-conjugated goat anti-human IgG (H+L) antibody (Li-COR Biosciences).

### Identification of the binding domain of human IgG using the ten fusion proteins

For the His pull-down assay, the human IgG Fab and Fc fragments (Calbiochem, Darmstadt, Germany) were incubated with the recombinant protein-bound resins as described above.

For the ELISA assay, ELISA plates were coated with the Fab and Fc fragments of human IgG. The ten fusion proteins were diluted, incubated and detected as described above.

### Competitive binding between host immune components and schistosomal ligands

To investigate the binding site between IgG and IgM on the parasite ligands, ELISA plates were coated with human IgG and 400 μg/ml isopyknic recombinant protein mixed with 400 μg/ml human IgM was added to the wells. The binding between human IgG and the His-tagged protein was detected using His-tagged mouse McAb and ALP-anti-mouse-IgG antibody at OD_405_ and compared with the wells lacking IgM.

To investigate the binding site of complement and human IgG on the parasite proteins, wells were coated with the same amount of human IgG or C1q (Calbiochem, Darmstadt, Germany) by weight (250 ng/well) and C1qBP was added to the wells in a series of dilutions ranging from 400 μg/ml to 6.25 μg/ml. Then, the interaction was detected as described above. Additionally, wells were coated with human C1q prior to the addition of 400 μg/ml of C1qBP mixed with various concentrations of human IgG (from 400 μg/ml to 12.5 μg/ml). Binding between C1q and C1qBP was detected as described above.

The statistical significance was determined from Student’s *t*-test for two-group comparisons using SPSS (Statistical Package for the Social Sciences) Statistics 17.0 software. The *P-value < 0.05, **P-value < 0.01 and ***P-value < 0.001 were considered significant.

### Relative quantification analysis

The expression levels of the ten proteins during various developmental stages of the *S. japonicum* parasites were examined using qRT-PCR according to a standard protocol. cDNA was extracted from eggs, cercariae, hepatic schistosomula (14 d), and adult male and female worms (42 d) according to a method described in previous reports^59^,^60^. The reactions were performed in technological triplicate on the 7300 Real-Time PCR system (Applied Biosystems, Carlsbad, USA) using Brilliant II SYBR Green QPCR Master Mix (Agilent Technologies, Santa Clara, USA) according to the manufacturer’s instructions. The 26S proteasome non-ATPase regulatory subunit 4 (PSMD4), which has been validated as a reliable reference gene for transcriptomic analysis of *S. japonicum*^56^, was employed as a control gene in the qRT-PCR analysis. The qRT-PCR primers were designed using Primer Express 3.0 software (Applied Biosystems) (Table 2). The relative expression level of each gene was analyzed using the software SDS 1.4 (Applied Biosystems).

## Additional Information

**How to cite this article**: Wu, C. *et al.* Non-immune immunoglobulins shield *Schistosoma japonicum* from host immunorecognition. *Sci. Rep.* **5**, 13434; doi: 10.1038/srep13434 (2015).

## Supplementary Material

Supplementary Dataset

Supplementary Information

## Figures and Tables

**Table 1:** Proteins associated with Ig-binding and host-parasite interactions in the immunoglobulin-binding proteomes of *Schistosoma japonicum.*

**Features**	**Number**
Annexin domain-containing protein	9
Ca^2+^ binding protein	25
Cathepsin	6
Complement binding protein	1
Diazepam-binding inhibitor	2
Dynein_light superfamily member	10
EF-hand domain-containing protein	9
EGF-like superfamily member	4
Immunogenic protein	18
Immunoglobulin domain-containing protein	16
Immunophilin	1
Laminin-related protein	3
LDLa superfamily member	3
Transmembrane protein	39
Ubiquitin-related protein	7
Zinc finger protein	12

**Table 2:** Summary information of the ten proteins tested for immunoglobulin binding.

**Protein Name**	Ig-bindingCapacity	*S. j* Protein DatabaseID	NCBI ProteinDatabase ID	**Features**	Gene CloningPrimers	Expression Vectorand Competentcells	**Primers of qRT-PCR**
Similar to immunophilin	IgG, IgM	gnl|lsbi|CPRT0000001567	AAW27121.1	Surface membrane protein, Immunophilin, Immunogenic protein	F:*GGATCC*GATGAACAAGGACGCAGT	pET-22b Rosetta(DE3)	F:GCATCTGCTGTAAAACAGGTTC
					R:*CTCGAG*CAGGGCAGACTTTTCATCATT		R:TCATTCACTACAACCCCATCCT
Sj20.8	IgG, IgM	gnl|lsbi|CPRT0000004432	AAP06272.1	EFh superfamily, Tegument antigen,Dynein_light superfamily,Ca^2+^ binding site	F:*GGATCC*GATGGAACCATTTGTTCAAGT	pET-22b BL21(DE3)	F:TGGCATGTTGTCATAGTTAGAGG
					R:*CTCGAG*TTCACCGGTCAATTCTTCGT		R: ATTCTTCGTCGGGTGTTCTCC
Sj31	IgG	gnl|lsbi|CPRT0000007256	CAX71564.1	Cathepsin,Immunogenic protein	F:*GGATCC*GATGCTGAACATCGCGTTTTGT	pET-22b TransB(DE3)	F: GGATTACTGGGTATTGCGTGG
					R:*CTCGAG*CGATTTTATTAGTCCAGCAGCT		R: TTTTGGAAATGGATAGGGTCG
Sj22.6	IgG, IgM	gnl|lsbi|CPRT0000007640	AAB52407.1	EFh superfamily,Membrane-associated antigen	F:*GGATCC*GATGGCAACTACTGAGTACAGATT	pET-22b BL21(DE3)	F: AGTTTTGTCGTGGATTCGGTC
					R:*CTCGAG*CGAAGACGGTGTTCGCCACAAT		R: CTTTGCCTTCCTTGTCTCTCTT
C1qBP	IgG	gnl|lsbi|CPRT0000007943	AAW26757.1	Complement binding protein	F:*GGATCC*ATGTCTCGATTTGTTGCTCGT	pET-28a Rosetta(DE3)	F: GAGGCTCATCCTGATTTGCG
					R:*CTCGAG*TTCTTTACAGTAATTCTGGAAT		R: GTGTCACCATCCGAGAGTTGTT
Sj-P40	IgG, IgM, IgE	gnl|lsbi|CPRT0000008913	AAW24545.1	Major egg antigen	F:*GGATCC*GATGGATAACTACAGAACAG	pET-22bBL21(DE3)	F: CATCGTCAACTTGGCATCCG
					R:*CTCGAG*AACTGTGCATAATAAT		R: CCTCCCATTGATACCATCCTTC
Tropomyosin	IgG, IgM	gnl|lsbi|CPRT0000009708	CAX72815.1	Immunogenic protein	F:*GGATCC*GATGGATGGAATTAAAAAG	pET-22b BL21(DE3)	F: GCTCGTCTTGAAGCAGCAGA
					R:*CTCGAG*ATAACCAGTAAGTTCTGCAAAAGT		R: GCTCTTTGTTCAGCAGCCTTTA
SJCHGC06760 protein	IgG, IgM	gnl|lsbi|CPRT0000009944	AAW25344.1	Annexin superfamily,Ca^2+^ binding site	F: *GGATCC*ATGGCTAAAGTTTCT	pET-28a Rosetta(DE3)	F: ATCGGATACCAGTGGGGATT
					R: *CTCGAG*TTCACCAAGTAGAACAC		R: GCATTGGCTACTGCTCTCATT
Annexin A13	IgG, IgM	gnl|lsbi|CPRT0000011452	CAX70810.1	Annexin superfamily,Ca^2+^ binding site	F:*GGATCC*ATGGGAAGATCTAAAATTC	pET-28a Rosetta(DE3)	F: GATGCTGGTGAAGCACGTCTA
					R:*CTCGAG*ACTCCATTCAGCACCAATT		R: CAATCGCTTGTATATGCCAAAC
Sjc23-LED	IgG	gnl|lsbi|CPRT0000000982	P27591.1	CD63 antigen,Tetraspanin	F:*GGATCC*GTACAAGGATAAAATCGATG	pET-22b BL21(DE3)	F: ATGACTGGTGCTCTGGAAAATC
					R:*CTCGAG*GTTGCGTTTTAAGAATGCACTAAAG		R: CCTGAACATAAACTTCTTGCCC

**Table 3:** Binding capacities of the ten proteins to human and animal immunoglobulins.

	HumanIgG	HumanIgM	Human IgE	HumanIgA	HumanIgD	Porcine IgG	BovineIgG
**Similar to immunophilin**	+	+	−	+	+	+	+
**Sj20.8**	+	+	−	+	+	+	+
**Sj31**	+	−	−	−	+	+	+
**Sj22.6**	+	+	−	+	+	+	+
**C1qBP**	+	−	−	+	+	+	+
**Sj-P40**	+	+	+	−	−	+	+
**Tropomyosin**	+	+	−	−	−	+	+
**SJCHGC06760 protein**	+	+	−	−	−	+	+
**Annexin A13**	+	+	−	−	−	+	+
**Sjc23-LED**	+	−	−	−	−	+	−
