# Supplementary material for: Non-immune immunoglobulins shield Schistosoma japonicum from host immunorecognition
Source: Sci Rep. 2015 Aug 24;5:13434. doi: 10.1038/srep13434 (PMC4547136; doi:10.1038/srep13434)

Non-immune immunoglobulins shield *Schistosoma japonicum* from host immunorecognition

Chuang Wu[1#], Nan Hou[1#], Xianyu Piao[1], Shuai Liu[1], Pengfei Cai[1], Yan Xiao[1], Qijun Chen[1, 2*]

[1]MOH Key Laboratory of Systems Biology of Pathogens, Institute of Pathogen Biology, Chinese Academy of Medical Sciences & Peking Union Medical College, Beijing 100730, P. R. China.

[2]Key Laboratory of Zoonosis, The Ministry of Education, Jilin University, Changchun 130062, P. R. China.

[#]With equal contribution

*Corresponding author

Contact information:

*QC: qijun.chen@ipbcams.ac.cn

Supplementary Information

Supplementary Figure 1 | Molecular function classification for the identified proteome of *S. japonicum* using gene ontology (GO) analysis. (**a**)**,** (**b**)**,** and (**c**) represent the results of functional classification of the proteins bound to human IgG, IgM and IgE, respectively.

Supplementary Figure 2 | Biological processes distributions for the detected proteins of *S. japonicum* using gene ontology (GO) analysis. (**a**)**,** (**b**)**,** and (**c**) are the analytic results of the proteins grouped according to biological process bound to human IgG (a), IgM (b) and IgE (c), respectively.

Supplementary Figure 3 | Binding of human IgG domains to the ten proteins in ELISA assays. The ELISA plate was coated with the Fab or Fc fragments prior to the addition of recombinant His-tagged protein dilutions ranging from 400 μg/ml to 6.25 μg/ml. All ten of the proteins bound to the Fc fragment, while only Sj31 slightly adhered to the Fab fragment.

Supplementary Data 1 | *Schistosoma japonicum* proteins that bind to non-immune human immunoglobulins.

Supplementary Table 1 | Description of the 4 genes encoding immunoglobulin-binding proteins that were differentially expressed in animal hosts during the adult stage.

***S. j* Protein Database ID**	**Protein Described in the Database**	**NCBI Protein Database ID**	**Ig-binding Capacity**	**ProbeSetID**	**Expression Quantity**			
					**C57/BL6 mouse**	**Balb/c mouse**	**Rabbit**	**Buffalo**
gnl|lsbi| CPRT0000000807	Dynein light chain LC8-type 2	AAX30103.1	IgE	CNUS0000096200.1	1057	661	947	1338
gnl|lsbi| CPRT0000002977	Diazepam binding inhibitor	CAX74960.1	IgE	CNUS0000098370.1	377	222	223	57
gnl|lsbi| CPRT0000006877	Basement membrane proteoglycan precursor	AAX24541.2	IgM	Sja_S23368570	749	705	287	415
gnl|lsbi| CPRT0000008978	Synaptic vesicular amine transporter (Monoamine transporter)	CAX69443.1	IgE	Sja_S53129181	1318	1737	2833	2233

Supplementary Figure 1

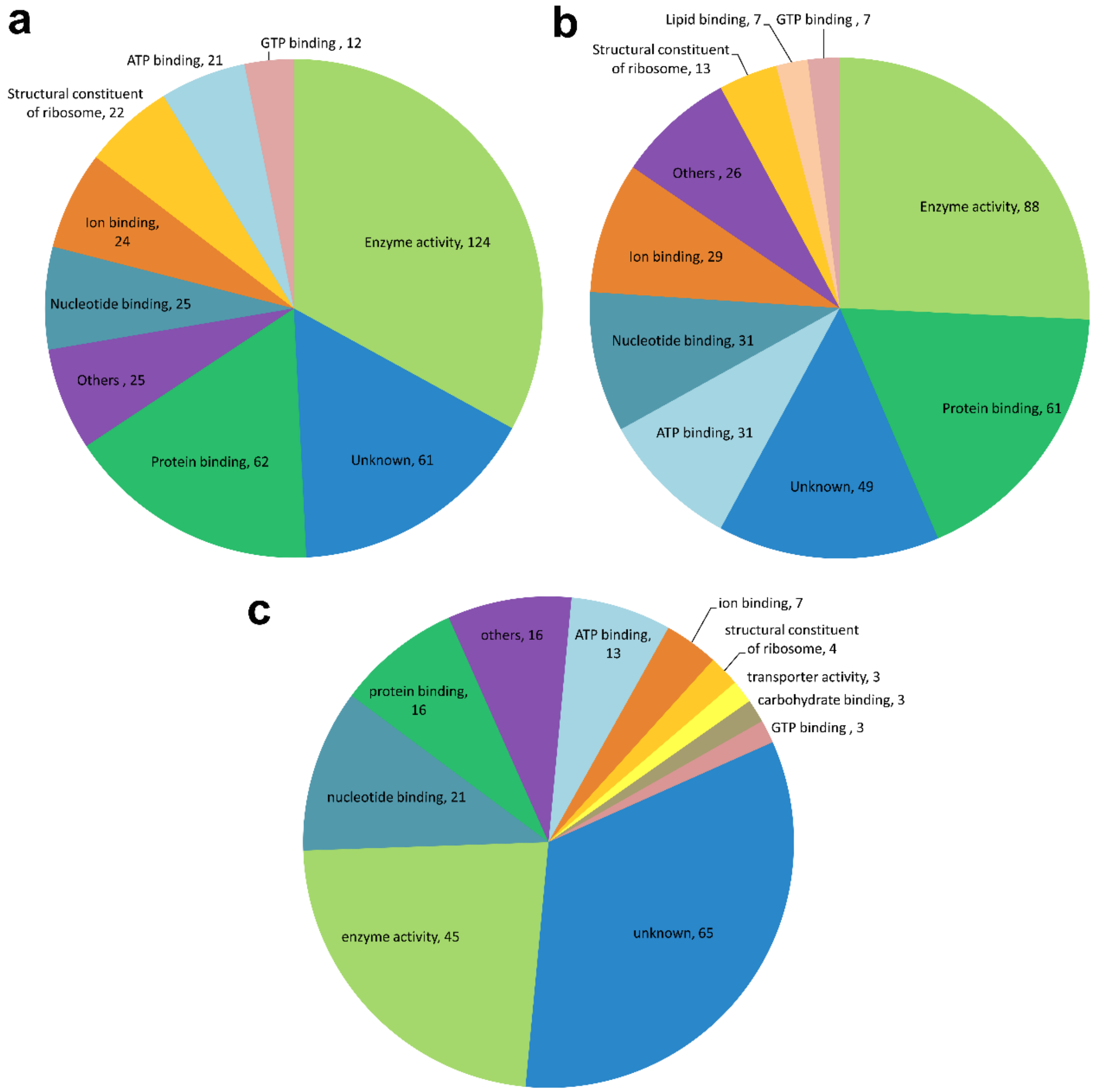

Supplementary Figure 2

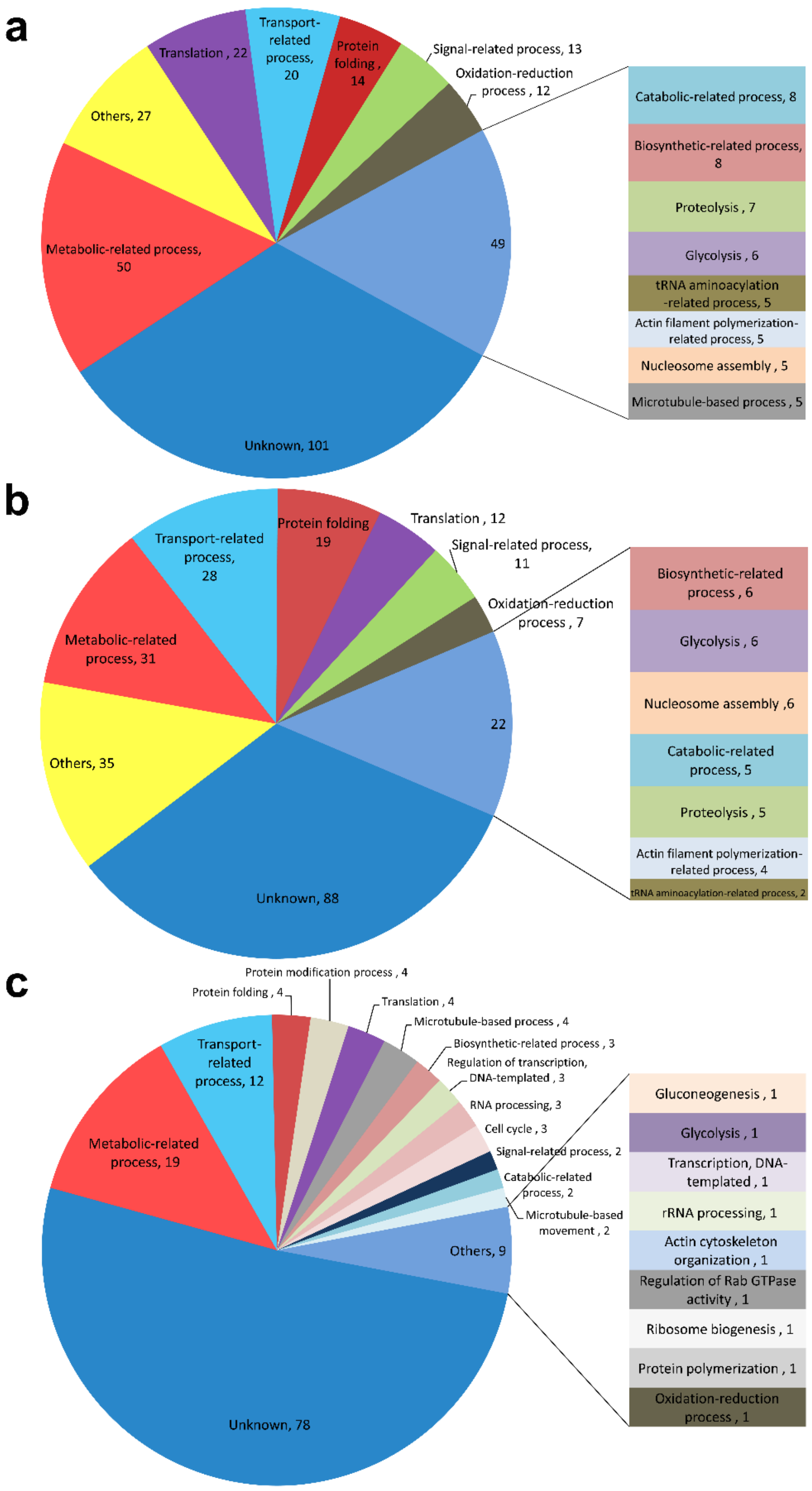

Supplementary Figure 3

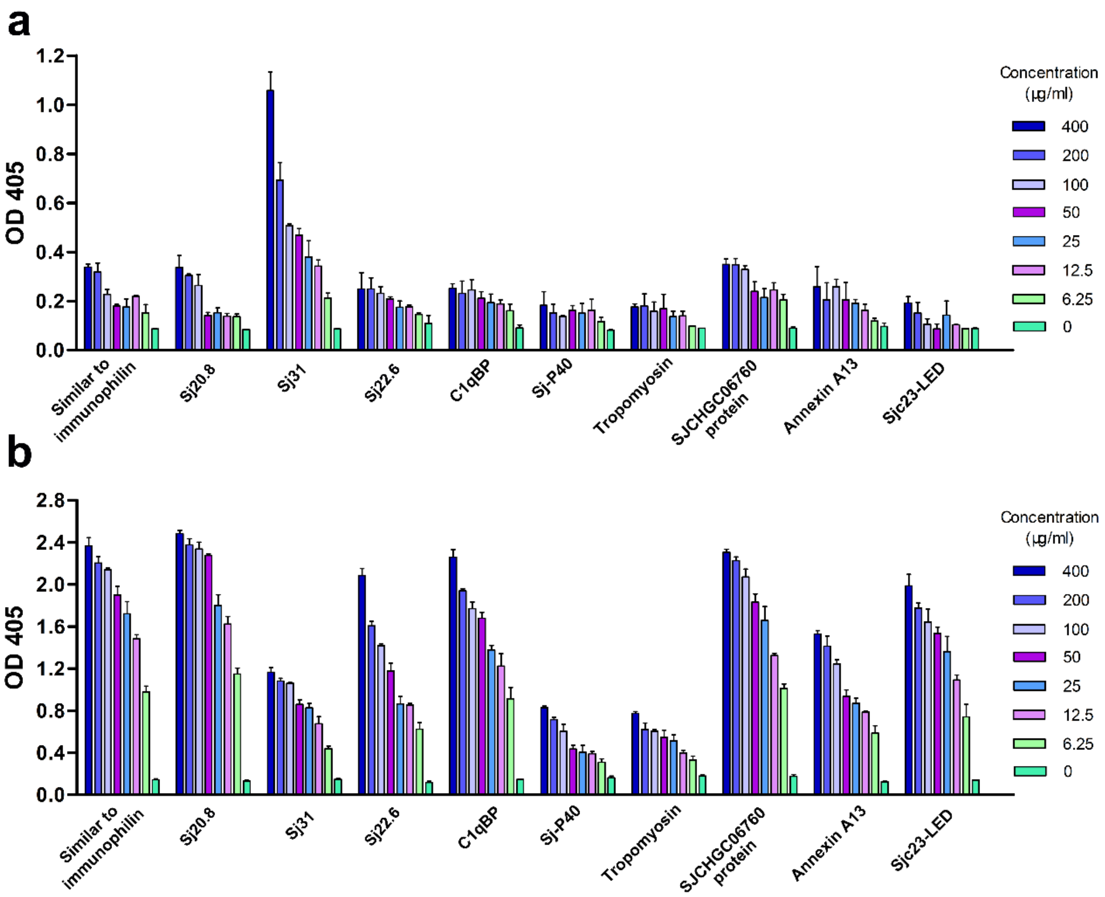

Supplement: Supplementary Information [file srep13434-s2.pdf]
